# Effect of clodronate-liposome injections on *Plasmodium falciparum* infection in neo-tropical primates *Saimiri sciureus*

**DOI:** 10.1186/1475-2875-13-S1-P23

**Published:** 2014-09-22

**Authors:** Janaiara A Cunha, Cesare Bianco-Junior, Marcelo P Machado, Pierre Druilhe, Leonardo JM Carvalho, Cláudio T Daniel-Ribeiro

**Affiliations:** 1 Laboratório de Pesquisas em Malária, Instituto Oswaldo Cruz, Fiocruz, Rio de Janeiro, Brazil; 2 Laboratório de Patologia, Instituto Oswaldo Cruz, Fiocruz, Rio de Janeiro, Brazil; 3 Vac-4-All iniciative, Paris, France

## Background

One of the constraints in the development of antimalarial vaccines is the limited availability of experimental models that reliably reproduce the infection and the immunopathology observed in human malaria. The best experimental models are Saimiri and Aotus neotropical primates, but they have as important limitations, the need for splenectomy for the expression of high and consistent parasitemias. The clodronate-liposome (CL) has been used to deplete macrophages in several experimental models and we tested the hypothesis that their use in Saimiri would allow the development of consistent *P. falciparum* parasitemias without the need for splenectomy.

## Methods

Two studies with non-splenectomized animals were performed. In the first study: six animals were divided in two groups receiving 1mL PBS or CL (5 mg/mL) and were infected at d0 with *P. falciparum* (FUP strain). In the second study: 14 animals were used in six groups - three uninfected controls groups with 2 animals each receiving 1 mL PBS, 0.5 mL or 1 mL CL and three groups infected at d0 and that received the same injections (PBS group with 2 animals and CL groups with 3 animals each one). In both studies the injections were performed intravenously twice weekly from d0. Follow-up was performed. Histological examination of spleen and liver was performed at the end of the study.

## Results

In the first study, animals receiving CL had high parasitemias requiring antimalarial treatment, whereas animals receiving PBS controlled the infection. In the second experiment, infected animals receiving 0.5 mL CL showed parasitemia higher than the other two groups. In all infected animals, the temperature was related to parasitemia. Hemoglobin and hematocrit concentration decreased reaching minimum values during or after clearance of the parasite. The animals tolerated the CL injections, but in some cases signs of liver toxicity were observed on histopathology. Animals receiving CL showed decreased iron staining in the spleen.

## Conclusions

Our results suggest that CL is able to promote higher parasitemias and could be an alternative to surgical splenectomy. Additional studies are needed to confirm the data.

